# Association between apolipoprotein E gene polymorphisms and the effects of high-intensity interval training on body composition in university students

**DOI:** 10.3389/fnut.2026.1769818

**Published:** 2026-03-26

**Authors:** Hao-Nan Chu, Wen-Wen Chu, Shan-Rong Xu, Yan Liu, Shu-Chen Liu, Teng Yao, Duo-Qi Zhou

**Affiliations:** School of Physical Education, Anqing Normal University, Anqing, Anhui, China

**Keywords:** APOE, body composition, high-intensity interval training, sensitivity, single nucleotide polymorphism

## Abstract

**Objective:**

This study examined the effects of APOE gene polymorphisms on body composition changes following high-intensity interval training (HIIT) in non-athletic Han Chinese university students from plain regions and identified genetic loci associated with HIIT sensitivity.

**Methods:**

A total of 236 Han Chinese undergraduates from non-physical education majors completed a 12-week HIIT program (three sessions/week). Body composition was assessed before and after the intervention. Genomic DNA from white blood cells was genotyped using Illumina chips. Single nucleotide polymorphism (SNP) quality control and association analyses with body composition indices were performed using PLINK (v1.09) and SPSS 25.0, applying linear regression and ANOVA with least significant difference (LSD) *post hoc* tests (*p* < 0.05).

**Results:**

(1) Of 22 initial APOE SNPs, five passed quality control; the rs405509 locus was associated with HIIT-induced changes in body composition. (2) The GG genotype at rs405509 was associated with higher baseline BMI overall and with higher baseline weight, BMI, and waist-to-hip ratio in females than the TT genotype. (3) After training, GG carriers showed greater reductions in overall body fat than GT/TT carriers (*p* < 0.05), and female GG carriers exhibited greater improvements in weight, body fat, and BMI. (4) In the Bonferroni *post hoc* analysis, compared with the TT group, the GG group showed significantly lower body weight (MD = −1.95, 95% CI: −3.374 to −0.533, *p* = 0.003), weight percentage (MD = −3.44, 95% CI: −6.070 to −0.819, *p* = 0.006), and BMI (MD = −0.70, 95% CI: −1.264 to −0.139, *p* = 0.009), while the difference in skeletal muscle percentage was not significant (*p* = 0.107). Compared with the GT group, the GG group also had significantly lower body weight, weight percentage, skeletal muscle percentage, and BMI (*p* = 0.02–0.03). No significant differences were observed between the GT and TT groups (all *p* > 0.05, with 95% CIs crossing zero). (5) The rs405509 locus was significantly associated with HIIT sensitivity for body fat (*p* = 0.01873).

**Conclusion:**

The rs405509 locus of the APOE gene is associated with body composition responses to HIIT, and female GG carriers show heightened responsiveness.

## 1 Introduction

Body composition serves as a crucial indicator for assessing human health, encompassing key metrics such as body weight and body fat percentage. Abnormal body composition is significantly associated with various metabolic disorders, including central obesity and type 2 diabetes (1, 2). Among these, body fat is the most closely monitored indicator, as it is widely recognized that excessive body fat is closely linked to increased morbidity and mortality rates (3). Moreover, fat accumulation in different locations leads to distinct metabolic issues. Excess visceral fat increases the risk of heart disease and obesity (4); Excessive body fat leads to insulin resistance, type 2 diabetes, and increased risk of cardiovascular disease (5). Currently, obesity rates among the Chinese population continue to rise, and chronic non-communicable diseases caused by overweight are major risk factors threatening the health of the Chinese people (6, 7). According to monitoring data from the Medium- and Long-Term Youth Development Plan (2016–2025), China’s youth population is facing prominent health issues such as increasing body weight, reduced basal metabolic rate, and abnormal accumulation of body fat (8).

Against this backdrop, HIIT has emerged as an effective strategy for addressing health issues among young adults due to its high time-efficiency ratio and significant effects on improving body composition and metabolic health (9, 10). Research has revealed that HIIT-related training outcomes are associated with single nucleotide polymorphisms (SNPs). However, there exists considerable variability in individual responses to exercise interventions, largely determined by genetic factors (11). Additionally, our previous research has identified that the rs694539, rs1941404, and rs6753096 loci in the NNMT and ADCY3 genes are associated with body composition sensitivity following HIIT training (12, 13).

Apolipoprotein E (ApoE) is a polymorphic protein that plays a central role in lipid metabolism, inflammatory regulation, and reverse cholesterol transport. The ApoE gene is a key regulator of blood lipid levels and obesity risk (14, 15). Its genetic polymorphisms (particularly the ε2/ε3/ε4 subtypes formed by rs429358 and rs7412) have been extensively reported to be associated with baseline lipid levels (16, 17). Different genotypes can influence the effects of exercise on lipid metabolism, leading to interindividual variations. The apolipoprotein E (ApoE) gene is located on chromosome 19 and contains two polymorphic sites (rs429358 and rs7412), giving rise to three alleles: E2, E3, and E4. These combine to form six genotypes: E2/E2, E2/E3, E2/E4, E3/E3, E3/E4, and E4/E4, corresponding to the ApoE2, ApoE3, and ApoE4 proteins, respectively; The distinction between these three proteins lies in amino acids at positions 112 and 158: ApoE2 has cysteine at both sites, ApoE3 has cysteine at one site and arginine at the other, while ApoE4 has arginine at both sites (18). However, current research primarily focuses on the effects of the classic ε2/ε3/ε4 haplotypes on lipid components and other factors. Notably, rs405509 is located within the APOE gene promoter region at position -219 T/G. This site modulates APOE gene expression by influencing transcription factor binding to the promoter, thereby linking it to lipid metabolism and multiple disease risks. The T allele exhibits only 60% of the transcriptional activity observed with the G allele (19, 20), potentially regulating downstream metabolic phenotypes. Although ample evidence demonstrates its association with blood lipid levels (16, 21), whether APOE influences individual responsiveness to exercise interventions—particularly HIIT—remains unknown.

Huang et al. (22) found that obesity is closely associated with dyslipidemia, and apolipoprotein E may influence obesity development through multiple mechanisms, including the inflammatory response, lipid metabolism, and energy expenditure. Zhang et al. (15) discovered that APOE deficiency exacerbates adipose tissue inflammation by activating the NOD-like receptor family, pyrin domain containing 3 (NLRP3) inflammasome, and that APOE expression levels are negatively correlated with human obesity development. Shatwan et al. (16) found that the APOE rs405509 locus is associated with high-density lipoprotein (HDL), with the GG genotype exhibiting higher HDL levels than the TT and GT genotypes, and is also linked to obesity. Furthermore, studies by Liu et al. (23) and Niimi et al. (24) both confirmed that APOE gene knockout (in mice and rabbits) leads to spontaneous hyperlipidemia and lipid deposition. Evidence also indicates that the ApoE gene participates in cholesterol reverse transport, a process crucial for maintaining lipid homeostasis in the body (25, 26). We examined a human adipose tissue database related to obesity (GEO accession: GSE9624). Results showed that compared to the normal-weight group, individuals in the obese group exhibited downregulated expression of ApoE in omental adipose tissue (OAT), along with significant differences in oxidative stress, inflammatory response, and lipid metabolism (27, 28). In summary, the APOE gene is implicated in lipid metabolism, inflammatory responses, and gene expression, and its polymorphisms may influence variations in training outcomes. This study aims to investigate the association between APOE gene polymorphisms and body composition as well as HIIT training sensitivity in Chinese university students after 12 weeks of high-intensity interval training. Although previous studies have examined the relationship between the APOE gene and lipid metabolism, the genetic mechanisms underlying its influence on body composition during exercise interventions particularly HIIT—remain unclear. This study is the first to explore the association between the APOE rs405509 locus and HIIT-induced improvements in body composition among Han Chinese university students, providing a genetic basis for personalized exercise prescriptions.

## 2 Study population and methods

### 2.1 Study population

A total of 236 not majoring in physical education Han Chinese undergraduate students were selected as subjects. Participants were recruited from Anqing Normal University, Jiangxi Normal University, Inner Mongolia Normal University, and Lanzhou City University (Table 1). All subjects were in good health with no harmful habits. Pre-test physical risk screening was conducted, and all participants provided informed consent. Subjects were instructed to refrain from medication intake, other physical training, and to maintain their usual dietary habits during the intervention period. This study has been approved by the Ethics Committee of Anqing Normal University and the Ethics Committee of Beijing Sport University. The ethical review approval numbers are 2018018H and 2024-YXLL-004, respectively.

**Table 1:** Overview of subject demographics.

Group	*n*	Age (year)	Height (cm)	Weight (kg)
Total	236	19.01 ± 1.14	166.18 ± 8.8	59.23 ± 11.6
Male	108	19.14 ± 1.2	173.4 ± 5.8	66.68 ± 11.48
Female	128	18.91 ± 1.08	160.09 ± 5.75	52.95 ± 7.14

### 2.2 Research methods

#### 2.2.1 High-intensity interval training protocol

**Figure 1:**
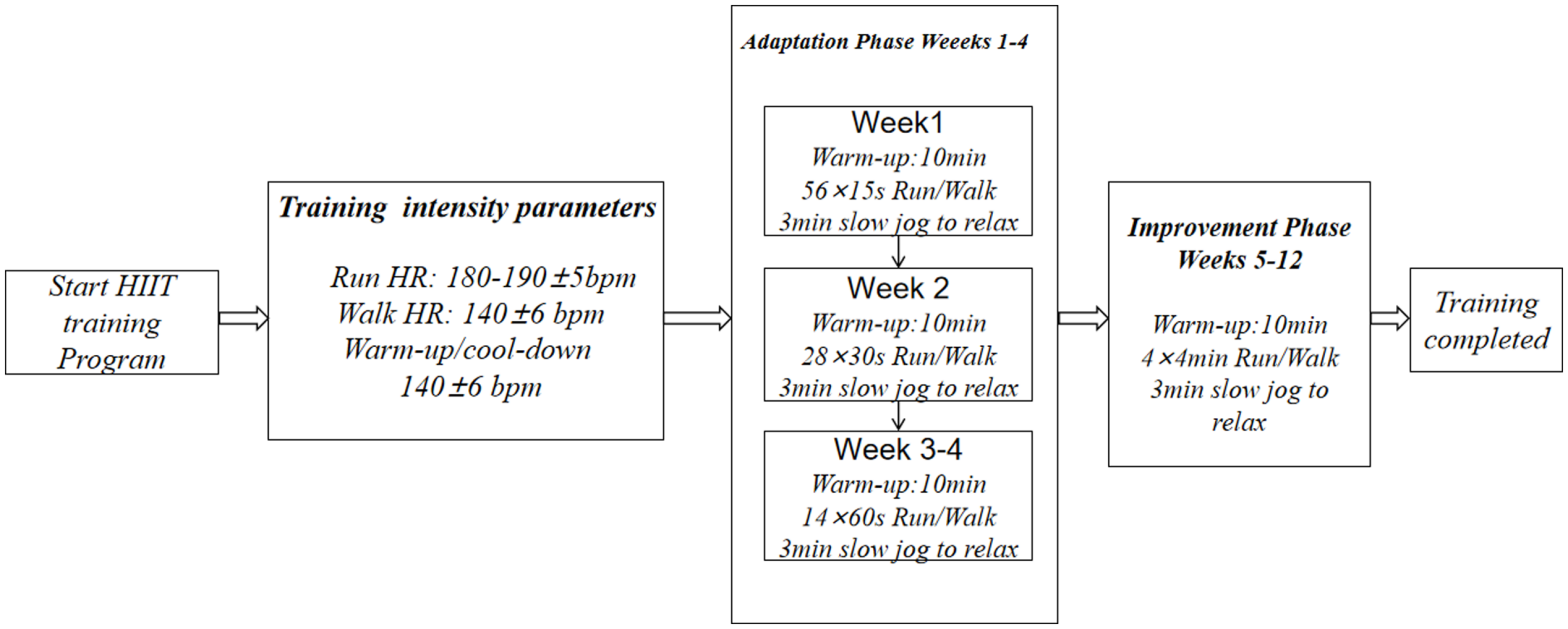
HIIT training program.

This study adopted the training protocol developed by Xu et al. (29). The protocol employed a phased progressive model lasting 12 weeks, with three training sessions per week, each lasting 28 min. The training cycle comprised two phases: an adaptation phase (Weeks 1–4) and an intensification phase (Weeks 5–12). Maximal heart rate (HRmax) was estimated using the formula HRmax = 220 − age. HIIT work intervals targeted 90–95% HRmax, with recovery intervals at 60–70% HRmax, consistent with high-intensity interval training guidelines. Adaptation Phase: The initial 4 weeks established a fitness foundation through progressive run-walk interval training. Week 1: Alternate 15-s runs (target heart rate zone: 180–190 ± 5 bpm, corresponding to 90–95% HRmax) with 15-s brisk walks (140 ± 6 bpm, corresponding to 70% HRmax). Week 2: Extend to 30-s run/walk intervals. Weeks 3–4 transition to 60-s and 120-s run-walk intervals, respectively. Maintain exercise intensity within the preset heart rate zone throughout. Progression Phase: Starting Week 5, adopt a standardized interval protocol: Begin with 240 s of continuous running at 90–95% HRmax intensity (heart rate: 180–190 ± 5 bpm), followed by 180 s of brisk walking at 70% HRmax intensity (heart rate: 140 ± 6 bpm), completing a 28-min training cycle. Each session begins with a 10-min slow jog warm-up. A Finnish-made Polar H10 heart rate monitor tracks physiological feedback in real-time throughout. Training is supervised by a dedicated professional team to ensure proper form, compliance with intensity guidelines and exercise safety (Figure 1).

#### 2.2.2 Body composition testing method

Testing was conducted using an InBody body composition analyzer (Model: 260, Manufactured in Shenzhen). Measured parameters included body weight, body weight percentage, skeletal muscle mass, skeletal muscle percentage, body fat mass, body fat percentage, body water, BMI, waist-to-hip ratio, fat-free mass, and basal metabolic rate. Body composition testing requires subjects to fast upon waking and remove all metal jewelry and mobile phones before testing. To ensure data accuracy, all measurements were performed by the same team of professionals.

#### 2.2.3 Gene extraction and genotyping

Blood samples were collected 24 h after the final training session, with subjects fasting for ≥10 h. Five milliliters of venous blood were collected using sterile vacuum tubes and immediately stored at −80 °C. The genomic DNA extraction process included: thawing samples, isolating leukocytes, treating them with a nuclear lysis buffer, and performing nucleic acid purification strictly following the standardized protocol of the TIANGEN DNA extraction kit. Genotyping was performed using the Illumina NovaSeq 6,000 high-throughput sequencing platform. Quality-controlled DNA samples were scanned and analyzed with the CGA whole-genome SNP chip, precisely capturing polymorphism information at target gene sites using whole-genome sequencing technology.

#### 2.2.4 Statistical methods

Phenotypes were tested for normal distribution using SPSS 26.0. The PLINK software (V1.09) was employed to screen and perform quality control on the obtained SNPs. Quality control criteria were: minimum allele frequency (MAF) > 0.05; SNP detection rate > 90%; sample detection rate >90%; H-W *p*-value >0.05 (Table 2). Selected SNP loci meeting these criteria were further analyzed in relation to body composition phenotypes. Linkage disequilibrium analysis of qualified SNP loci was performed using Haploview software, with criteria: MAF > 0.05, *r*^2^ ≥ 0.8 (30). Intergroup differences were assessed using a one-way ANOVA with LSD *post hoc* comparisons at a *p* < 0.05 significance level, and were adjusted by Bonferroni correction. Results are presented as mean ± SD.

**Table 2:** The APOE gene SNPs included in the study after quality control screening.

CHR	SNP	Loci	Major allele	Minor allele	MAF	Genotype frequency	H-WP value
**19**	Rs405509*	**45,408,836**	**G**	**T**	**0.2754**	**127/88/21**	**0.328**
19	Rs7412	45,412,079	T	C	0.1017	191/42/3	0.7165
19	rs150375400	45,409,482	G	A	0.02331	225/11/0	0.6112
19	Rs769449	45,410,002	A	G	0.06992	203/33/0	1
19	rs190853081	45,412,340	A	G	0.004237	234/2/0	1

CHR, chromosome; SNP, single nucleotide polymorphism; MAF, minor allele frequency; H-W, Hardy–Weinberg test. *Site in italics and bold is site with significant differences in association analysis.

#### 2.2.5 Factor adjustment

This study employed the linear regression model in PLINK (V1.09) software to analyze the association between APOE gene SNP loci and body composition indicators using the additive genetic effect (ADD) model. By incorporating gender, height, age, and baseline body composition measurements as a covariate matrix, this method quantifies the independent genetic effects of each SNP locus on the target phenotype through regression equations. This approach controls for covariate interference while assessing the contribution of genetic variation to body composition phenotype variation, ensuring the statistical validity of association analysis results.

## 3 Results

### 3.1 APOE gene SNP information

Gene chip scanning identified 22 SNP loci in the APOE gene. Quality control and screening of these 22 SNP loci were performed using PLINK, ultimately selecting 5 loci for association analysis (Table 2). Linear regression model analysis revealed that the rs405509 locus influences body fat, demonstrating a significant correlation with body composition (*p* < 0.05). Linkage disequilibrium analysis of the SNP sites using Haploview software revealed no sites linked to rs405509. Therefore, further statistical analysis was conducted on the rs405509 site in relation to pre-HIIT composition and HIIT sensitivity.

### 3.2 Association analysis of APOE gene polymorphisms and body composition

#### 3.2.1 Association analysis of APOE gene polymorphisms and HIIT precursor components

Univariate analysis of variance (ANOVA) was conducted to examine the association between the rs405509 locus and baseline body composition parameters (Table 3). Results revealed significant associations between rs405509 and overall body weight percentage and BMI prior to HIIT (*p* < 0.05). Compared with subjects with GT/TT genotypes, those with the GG genotype exhibited higher baseline body weight percentage and BMI. By gender, rs405509 was associated with body weight, BMI, and waist-to-hip ratio in female subjects (*p* < 0.05). Female subjects with GG/GT genotypes exhibited higher baseline values than those with the TT genotype, suggesting that G allele carriers have higher baseline values. The rs405509 locus showed no significant association with any HIIT precursor component indicators in male subjects (*p* > 0.05) (Table 3).

**Table 3:** The association between rs405509 polymorphisms and the pre-HIIT body composition.

Body composition	Rs405509					
	Group	*n*	GG (21)	GT (88)	TT (127)	*P*
Weight	Total	236	63.03 ± 12.25	59.9 ± 12.27	57.62 ± 11.05	0.091
	Male	108	68.6 ± 11.99	68.15 ± 12.14	64.75 ± 11.18	0.299
	Female	128	57.97 ± 10.57	52.69 ± 6.54	51.82 ± 6.73	0.030^*^
Weight%	Total	236	103.65 ± 15.15	98.38 ± 12.96	96.09 ± 12.79	0.040^*^
	Male	108	102.73 ± 15.9	102.1 ± 15.3	97.72 ± 13.34	0.268
	Female	128	104.49 ± 15.16	95.14 ± 9.55	94.77 ± 12.27	0.036^*^
Skeletal muscle	Total	236	25.97 ± 6.05	25.24 ± 6.35	24.15 ± 5.73	0.257
	Male	108	30.81 ± 4.9	30.88 ± 4.41	29.42 ± 4.1	0.222
	Female	128	21.56 ± 2.59	20.32 ± 2.5	19.85 ± 2.14	0.068
Skeletal muscle%	Total	236	95.74 ± 7.87	94.17 ± 9.09	92.03 ± 8.06	0.066
	Male	108	94.99 ± 10.51	96.75 ± 10.36	92.83 ± 9.34	0.154
	Female	128	96.42 ± 4.83	91.92 ± 7.2	91.37 ± 6.85	0.079
Body fat	Total	236	16.12 ± 6.81	14.1 ± 5.43	13.65 ± 5.03	0.148
	Male	108	13.93 ± 6.36	13.2 ± 6.61	12.23 ± 5.23	0.585
	Female	128	18.11 ± 6.86	14.89 ± 4.04	14.8 ± 4.58	0.084
Body fat%	Total	236	25.29 ± 8.32	23.62 ± 7.31	23.71 ± 7.22	0.629
	Male	108	19.73 ± 6.48	18.67 ± 6.26	18.31 ± 4.91	0.756
	Female	128	30.34 ± 6.45	27.94 ± 5.12	28.1 ± 5.65	0.417
Body moisture	Total	236	34.4 ± 7.21	33.58 ± 7.63	32.24 ± 6.91	0.254
	Male	108	40.12 ± 5.85	40.31 ± 5.4	38.53 ± 5.05	0.232
	Female	128	29.2 ± 3.29	27.71 ± 3.05	27.12 ± 2.65	0.071
LBW	Total	236	46.91 ± 9.85	45.8 ± 10.42	43.97 ± 9.41	0.256
	Male	108	54.67 ± 8.1	54.96 ± 7.44	52.52 ± 6.94	0.236
	Female	128	39.86 ± 4.46	37.8 ± 4.16	37.01 ± 3.63	0.072
BMI	Total	236	22.44 ± 3.18	21.36 ± 2.93	20.82 ± 2.79	0.044^*^
	Male	108	22.61 ± 3.51	22.46 ± 3.37	21.5 ± 2.94	0.268
	Female	128	22.28 ± 3.02	20.4 ± 2.09	20.27 ± 2.54	0.040^*^
WHR	Total	236	0.86 ± 0.05	0.84 ± 0.04	0.83 ± 0.04	0.108
	Male	108	0.85 ± 0.05	0.84 ± 0.05	0.84 ± 0.05	0.899
	Female	128	0.86 ± 0.06	0.83 ± 0.02	0.83 ± 0.03	0.010^*^
BM	Total	236	1383.43 ± 212.67	1359.19 ± 225.16	1319.85 ± 203.1	0.256
	Male	108	1557.1 ± 160.48	1504.32 ± 149.67	1528.68 ± 156.84	0.234
	Female	128	1231.09 ± 96.35	1186.55 ± 90.13	1169.64 ± 78.42	0.073

Weight%: (body weight/ideal body weight) × 100%; Skeletal muscle%: (skeletal muscle/ideal skeletal muscle) × 100%; BMI, body mass index; WHR, waist-to-hip ratio; LBW, lean body weight; BM, basal metabolism. * indicates a significant difference in the test results (*P* < 0.05).

#### 3.2.2 APOE gene polymorphism and body composition in HIIT sensitivity

Following training, GG genotype subjects exhibited a significantly greater overall reduction in body fat compared to GT/TT genotype individuals (*p* < 0.05) (Table 4). GG genotype female subjects demonstrated more pronounced decreases in weight and BMI changes post-training (*p* < 0.05), with body fat showing a marginally significant trend (*p* = 0.053). Male subjects showed a similar trend in body fat metrics but the differences were not statistically significant (*p* > 0.05). Bonferroni-adjusted *post hoc* comparisons indicated that the GG genotype differed significantly from TT, showing lower body weight (MD = −1.95, 95% CI: −3.374 to −0.533, *p* = 0.003), weight percentage (MD = −3.44, 95% CI: −6.070 to −0.819, *p* = 0.006), and BMI (MD = −0.70, 95% CI: −1.264 to −0.139, *p* = 0.009), whereas skeletal muscle percentage did not differ (*p* = 0.107). Relative to GT, the GG group also had significantly lower body weight, weight percentage, skeletal muscle percentage, and BMI (*p* = 0.02–0.03). No significant differences were found between GT and TT (all *p* > 0.05; 95% CIs included zero) (Table 5). To eliminate the influence of factors such as gender on HIIT training effects, we conducted linear regression analysis (Linear) after controlling for height, gender, age, and initial body composition phenotype values as covariates. Under the effect of ADD, we found a significant association between the rs405509 locus and HIIT sensitivity to body fat (*p* = 0.01873).

**Table 4:** The rs405509 polymorphism and body composition sensitivity to HIIT.

Body composition	Rs405509								
	Group	*n*	GG (*n* = 21)	GT (*n* = 88)	TT (*n* = 127)	*P*	Test	*β*	*P*
Weight	Total	236	−0.62 ± 1.87	−0.25 ± 2.49	0.27 ± 2.15	0.105	ADD	−0.4437	0.04512^*^
	Male	108	0.02 ± 1.81	−1.06 ± 2.77	−0.3 ± 2.51	0.268			
	Female	128	−1.21 ± 1.81	0.46 ± 1.99	0.74 ± 1.67	0.005^**^			
Weight%	Total	236	−1.14 ± 3.38	−0.25 ± 3.92	0.43 ± 3.49	0.125	ADD	−0.6749	0.05847
	Male	108	0.05 ± 2.81	−1.5 ± 4.04	−0.54 ± 3.64	0.337			
	Female	128	−2.23 ± 3.6	0.84 ± 3.5	1.22 ± 3.18	0.008^**^			
Skeletal muscle	Total	236	0.27 ± 0.86	0.45 ± 0.89	0.48 ± 0.91	0.610	ADD	−0.07172	0.4245
	Male	108	0.51 ± 1.15	0.3 ± 0.92	0.36 ± 1.11	0.840			
	Female	128	0.05 ± 0.42	0.58 ± 0.86	0.57 ± 0.69	0.078			
Skeletal muscle%	Total	236	1.03 ± 3.15	2.25 ± 3.13	1.81 ± 3.07	0.241	ADD	−0.051	0.8707
	Male	108	2.32 ± 2.64	1.81 ± 2.68	1.53 ± 3.25	0.712			
	Female	128	−0.14 ± 3.24	2.63 ± 3.47	2.04 ± 2.92	0.036^*^			
Body fat	Total	236	−0.98 ± 1.5	−0.87 ± 1.84	−0.37 ± 1.34	0.037^*^	ADD	−0.3552	0.01873^*^
	Male	108	−0.77 ± 1.19	−1.45 ± 1.88	−0.76 ± 1.34	0.091			
	Female	128	−1.17 ± 1.77	−0.37 ± 1.67	−0.05 ± 1.25	0.053^#^			
Body fat%	Total	236	−1.23 ± 1.85	−1.25 ± 2.11	−0.7 ± 1.82	0.102	ADD	−0.3528	0.06554
	Male	108	−1.24 ± 1.67	−1.61 ± 1.67	−1.03 ± 1.69	0.251			
	Female	128	−1.23 ± 2.09	−0.93 ± 2.4	−0.43 ± 1.88	0.307			
Body moisture	Total	236	0.2 ± 0.98	0.39 ± 1.08	0.41 ± 1.1	0.733	ADD	−0.06093	0.5747
	Male	108	0.51 ± 1.28	0.21 ± 1.14	0.26 ± 1.34	0.800			
	Female	128	−0.07 ± 0.51	0.54 ± 1.02	0.52 ± 0.86	0.109			
LBW	Total	238	0.36 ± 1.39	0.62 ± 1.48	0.64 ± 1.51	0.712	ADD	−0.08846	0.5533
	Male	108	0.79 ± 1.8	0.39 ± 1.55	0.46 ± 1.84	0.805			
	Female	128	−0.04 ± 0.75	0.83 ± 1.4	0.79 ± 1.18	0.101			
BMI	Total	236	−0.24 ± 0.72	−0.06 ± 0.86	0.08 ± 0.76	0.156	ADD	−0.1381	0.07489
	Male	108	−0.01 ± 0.63	−0.34 ± 0.9	−0.13 ± 0.8	0.361			
	Female	128	−0.45 ± 0.77	0.18 ± 0.76	0.79 ± 1.18	0.012^*^			
WHR	Total	236	−0.01 ± 0.03	−0.02 ± 0.03	−0.01 ± 0.02	0.157	ADD	−0.0037	0.1099
	Male	108	−0.02 ± 0.02	−0.03 ± 0.02	−0.02 ± 0.02	0.092			
	Female	128	−0.01 ± 0.04	−0.01 ± 0.02	0 ± 0.02	0.581			
BM	Total	236	7.57 ± 29.57	13.38 ± 31.99	13.9 ± 32.53	0.702	ADD	−2.101	0.514
	Male	108	16.6 ± 38.7	8.32 ± 33.43	10.04 ± 39.6	0.820			
	Female	128	−0.64 ± 15.67	17.79 ± 30.34	17.04 ± 25.26	0.105			

Weight%: (body weight/ideal body weight) × 100%; Skeletal muscle%: (skeletal muscle/ideal skeletal muscle) × 100%; BMI, body mass index; WHR, waist-to-hip ratio; LBW, lean body weight; BM, basal metabolism; ADD, additive effect. */** indicates a significant difference in the test results (*P* < 0.05 or *p* < 0.01). # indicates edge prominence.

**Table 5:** Effect of Rs405509 on women after training following *post hoc* Bonferroni correction.

Body composition	Rs405509					
	Group		*M* ± SD	*P*	95%CI	*ω* ^2^
Weight	GG (*n* = 11)	GG/GT	−1.67 ± 0.60	0.02*	−3.136 ~ −0.202	0.067
	GT (*n* = 47)	GG/TT	−1.95 ± 0.59	0.003**	−3.374 ~ −0.533	
	TT (*n* = 70)	GT/TT	0.28 ± 0.34	>0.999	−0.541 ~ 1.111	
Weight%	GG (*n* = 11)	GG/GT	−3.06 ± 1.12	0.021*	−5.775 ~ −0.352	0.06
	GT (*n* = 47)	GG/TT	−3.44 ± 1.08	0.006**	−6.070 ~ −0.819	
	TT (*n* = 70)	GT/TT	0.38 ± 0.63	>0.999	−1.146 ~ 1.908	
Skeletal muscle%	GG (*n* = 11)	GG/GT	−2.76 ± 1.06	0.03*	−5.329 ~ −0.199	0.037
	GT (*n* = 47)	GG/TT	−2.17 ± 1.02	0.107	−4.658 ~ 0.309	
	TT (*n* = 70)	GT/TT	−0.59 ± 0.60	0.972	−2.033 ~ 0.855	
BMI	GG (*n* = 11)	GG/GT	−0.63 ± 0.24	0.029*	−1.209 ~ −0.048	0.053
	GT (*n* = 47)	GG/TT	−0.70 ± 0.23	0.009**	−1.264 ~ −0.139	
	TT (*n* = 70)	GT/TT	0.07 ± 0.13	>0.999	−0.254 ~ 0.400	

Weight%: (body weight/ideal body weight) × 100%; Skeletal muscle%: (skeletal muscle/ideal skeletal muscle) × 100%; BMI, body mass index; ADD, additive effect. */** indicates a significant difference in the test results (*P* < 0.05 or *P* < 0.01).

## 4 Discussion

### 4.1 Association analysis of APOE gene polymorphisms and body composition

Abnormal changes in body composition indicators increase the risk of non-communicable diseases, and these indicators can predict the occurrence of such diseases to a certain extent (30, 31). Studies indicate that blood lipids confound body fat, with body fat showing a positive correlation with triglycerides and total cholesterol. Abnormal blood lipid levels in humans are closely associated with the development of obesity (28). Tejedor et al. (21) found that the APOE gene variants rs7412 and rs429358 in middle-aged Caucasian men were strongly associated with changes in triglycerides and total cholesterol. Furthermore, the rs7412 locus of the APOE e2 subtype exhibited a dependent association with adult waist circumference and BMI. TOMM40-APOE is a pair of genes that are tightly linked in the genome and functionally interrelated, jointly regulating cellular lipid metabolism and energy production. Zhang et al. (32) conducted a genome-wide association study (GWAS) and found that in the Chinese population, the TOMM40-APOE (Translocase of Outer Mitochondrial Membrane 40-Apolipoprotein E) gene variant rs157580 was associated with reduced LDL-C and TC levels, as well as elevated TG levels. Studies indicate differences between Chinese and European populations in G allele frequency (0.22/0.14) and TG influence (0.14/0.30) (33).

Experimental studies by Komurcu-Bayrak et al. (34) demonstrated that the rs405509 locus of the APOE gene exhibits a negative correlation with insulin resistance (*p* < 0.05), and this association remained significant after adjusting for covariates such as age and gender. Furthermore, evidence from animal models provides additional support for APOE’s direct role in metabolic regulation: Knockout of the apoE gene in mice (apoE−/− mice) leads to significantly elevated serum cholesterol and triglyceride levels, along with spontaneous development of insulin resistance and visceral obesity (35). This model clearly demonstrates that loss of APOE gene function triggers lipid metabolism disorders and obesity. Previous studies have indicated that rs405509 may affect transcriptional activity; however, this allele-specific molecular effect may not be directly reflected in skeletal muscle percentage, a complex phenotype shaped by multiple determinants. In addition to skeletal muscle mass itself, skeletal muscle percentage is influenced by fat mass, overall body weight, age, sex, lifestyle, and other genetic factors (36). Furthermore, the effects of functional variants on complex traits are frequently modified by genetic background, environmental exposures, and intermediate biological processes (37). As a result, the phenotypic manifestation may not necessarily exhibit a monotonic pattern across genotypes.

The above studies indicate that the influence of APOE gene SNP loci on body composition holds profound biological significance. Through univariate analysis, this study investigated the association between the APOE gene rs405509 locus and body composition components, revealing significant correlations with weight, BMI, and other measures—consistent with prior research. A large-scale Indian study also identified an association between the APOE gene and obesity (38). However, a large-scale European genome-wide association study (GWAS) failed to detect a broad association between APOE genotypes and human obesity (39). Therefore, we speculate that differences in ethnic populations, regional variations in SNP loci, genetic effect disparities, and frequency variations may contribute to the inconsistencies in results.

### 4.2 Association analysis of APOE gene polymorphisms and body composition with HIIT sensitivity

This study found that after training, participants carrying the G allele at the rs405509 locus of the APOE gene demonstrated greater improvements in body fat (*p* < 0.05). Female participants with the GG genotype exhibited a more pronounced post-training decline in weight and BMI, with greater training effects (*p* < 0.05). A marginally significant reduction in body fat was observed in GG-type female participants (*p* = 0.053). No statistically significant differences were observed in the male group. This suggests that body composition at the APOE gene rs405509 locus may be associated with HIIT sensitivity. Studies on exercise training and the ACE gene indicate that factors such as gender and individual exercise capacity inherently influence the effects of exercise training (40). Studies on the APOE, SCARB1, and PPARα genes in lipid research have also identified gender differences, indicating that sex may influence exercise training outcomes (41). We employed linear regression models to correct for the effects of gender and other factors on phenotypes. After correction, this study found that the APOE gene rs405509 locus was significantly associated with HIIT training effects on body fat. Female subjects with the GG genotype demonstrated greater improvements in body weight, body fat, and BMI than those with the TT/GT genotypes. This suggests that the APOE rs405509 locus influences body composition sensitivity to HIIT, with the G allele conferring enhanced response in females. Based on the literature review, the proposed mechanism is as follows:

Lipid Metabolism Regulatory Pathways: (1) Multiple studies suggest that APOE gene polymorphisms may influence obesity regulation by affecting lipid metabolism. APOE serves as a key ligand for the Low-Density Lipoprotein Receptor (LDLR) and Low-Density Lipoprotein Receptor-Related Protein (LRP), participating in hepatic uptake of chylomicron (CM) and very low-density lipoprotein (VLDL) remnants. It also functions as a cofactor for lipoprotein lipase (LPL) in VLDL metabolism, participating in the metabolism of chylomicron (CM) and very low-density lipoprotein (VLDL) remnants, and in the breakdown of triglycerides in VLDL. It influences the formation of IDL and LDL. Different APOE genotypes exhibit variations in receptor affinity, thereby exerting distinct effects on lipid metabolism efficiency (14, 22). A study found that carriers of the APOE E4 allele among obese children exhibited elevated triglycerides (TG), low-density lipoprotein cholesterol (LDL-C), and body mass index (BMI), along with reduced high-density lipoprotein cholesterol (HDL-C). This effect synergized with the leptin receptor gene (LEPR) to influence lipid metabolism (42). A rat study also demonstrated reduced LPL activity in APOE knockout mice, with APOEE3 supplementation restoring activity, whereas APOEE4 showed limited efficacy, suggesting functional insufficiency of the E4 allele (43). Concurrently, additional evidence indicates that ApoE^−^/^−^ knockout mice exhibit elevated LDL-C, reduced HDL-C, diminished antioxidant capacity, and insulin resistance (44, 45).

TOMM40-APOE is a pair of tightly linked genes on the genome. Evidence indicates that exercise itself cannot alter these genes, but it can enhance mitochondrial function by activating pathways such as PGC-1α which upregulates the expression of mitochondrial-related genes like TOMM40, thereby improving lipid metabolism and obesity (46)^.^ Research in Taiwan has revealed that the T allele at this locus increases chromatin accessibility by elevating H3K27ac histone modification levels, thereby suppressing APOE gene transcription. This effect significantly impacts adipocyte differentiation and lipid storage, particularly in adipose tissue (28). Related reports indicate that this regulation exhibits tissue specificity and sexual dimorphism, with the APOE E4 allele showing a sex-specific association with metabolic syndrome, increasing risk only in females (47). Another report, based on gene and pathway analysis, revealed significant differences in transcription between females and males in fatty acid and amino acid pathways (48). It is speculated that this may be related to the synergistic regulation of the APOE promoter by estrogen receptors (28, 38, 43). Therefore, it is hypothesized that different genotypes and genders may be associated with lipid metabolism through alterations in APOE-receptor binding, which could in turn be related to the efficacy of HIIT in improving body composition. The potential mechanisms underlying these associations warrant further investigation.

(2) Inflammation and Oxidative Stress Mechanisms: A study reports that endogenous adipocyte ApoE participates in white fat browning, beige fat differentiation, and thermogenesis regulation: APOE4 reduces UCP1 activity, inhibits thermogenesis and energy expenditure, thereby promoting obesity (28). Furthermore, APOE E4 increases secretion of inflammatory mediators such as IL-6 and TNF-*α* in adipose tissue, inducing chronic inflammation via the JAK–STAT pathway and disrupting the balance of lipolysis (49, 50). The APOE gene also serves as an upstream regulator of AMPK activity. Its deficiency leads to reduced AMPK activity and autophagy levels, exacerbating inflammation and oxidative stress (51). HIIT activates the AMPK/SIRT1 pathway, enhances PGC-1α-mediated UCP1 transcription, and promotes thermogenesis. Concurrently, it modulates the miRNA expression profile in adipose tissue, relieves UCP1 inhibition, and improves inflammatory status, insulin sensitivity, and LPL activity (50, 52, 53). We propose a mechanistic hypothesis: the rs405509 G allele may suppress NLRP3 inflammasome activation in adipose tissue by maintaining higher APOE expression, thereby alleviating chronic inflammatory states. This places G allele carriers in a “low-inflammation” state more conducive to exercise-induced metabolic improvements, rendering them more sensitive to the anti-inflammatory and metabolic benefits of HIIT.

(3) From the perspective of APOE gene expression, it was found that the rs405509 site of the APOE gene is a promoter single nucleotide polymorphism (SNP) that affects APOE gene expression (54). Reports indicate that the APOE gene G allele is associated with altered blood lipid levels (38). Furthermore, studies have found that LDL and triglyceride levels are higher in GG allele E3/E3 subjects compared to TT allele subjects, and these findings hold true in the general population (54, 55). Additionally, the APOE promoter region contains polymorphic sites such as rs449647 and rs7259620. Studies in the Kazakh population of Xinjiang indicate that while these variants are not directly associated with cardiovascular disease or obesity risk, these loci can form haplotypes such as G-rs449647/A-rs7259620, which synergistically enhance APOE expression in adipose tissue and improve insulin sensitivity (56). Different APOE genotypes exert varying effects on BMI regulation (57). Research has found that the APOE rs429358 polymorphism is associated with a lower BMI in older adults (58). Meanwhile, in experiments with adult mice, gender differences were observed in the specific effects of APOE genotype expression (59). In summary, this further supports the role of this site in regulating lipid metabolism and body composition by modulating APOE gene expression, thereby influencing individual responses to exercise interventions.

(4) Research has demonstrated that under inflammatory stimulation, microglia with the APOE4/4 genotype induce the PI3K/AKT/mTORC signaling pathway (60, 61) and the PI3K/Akt pathway may also exert isoform-dependent effects in relation to ApoE (62, 63). Given the intermittent high-intensity and recovery pattern of HIIT, its effects on body weight, BMI, lean mass, and fat mass may be partly related to dynamic changes in energy stress, nutrient availability, and mTOR signaling, which has been implicated in autophagy, protein synthesis, and metabolic adaptation (64, 65). These mechanisms may partly explain the observed body composition changes, but because the present study did not directly assess pathway-related molecular markers, such interpretations remain speculative and require further confirmation.

This study explored the association between the APOE rs405509 polymorphism and HIIT-induced changes in body composition, aiming to identify potential genetic markers related to individual variability in training response. The rs405509 locus showed an association with differential HIIT effects on adiposity-related outcomes, and female participants carrying the GG genotype tended to exhibit greater responsiveness to training. These findings may offer a reference for the development of individualized exercise interventions in obese populations, particularly women. Additionally, the present results may serve as a reference for understanding APOE-related exercise responses in the Chinese Han population. Several limitations should be acknowledged. First, the absence of a sedentary or non-exercise control group limits our ability to fully exclude natural temporal influences, particularly given the multicenter recruitment from three universities with different climatic conditions and dietary habits. Second, dietary intake was not strictly controlled during the intervention period, which may have affected internal validity. Third, maximal heart rate was estimated using the 220 – age formula, which, although practical, may introduce individual error in exercise intensity prescription. Finally, the relatively small sample size, especially for the GG genotype subgroup, may have limited statistical power and generalizability. Therefore, these findings should be interpreted with caution and confirmed in future studies.

## Data Availability

The raw data supporting the conclusions of this article will be made available by the authors, without undue reservation.

## References

[1] Blüher M. An overview of obesity-related complications: the epidemiological evidence linking body weight and other markers of obesity to adverse health outcomes. Diabetes Obes Metab. (2025) 27:3–19. doi: 10.1111/dom.16263, 40069923 PMC12000860

[2] Mondal S Gargari P Bose C Garg MK Chowdhury S Mukhopadhyay S. Abnormal body composition increases the cardiometabolic risk in adolescents and young adults with turner syndrome. Endocr Pract. (2024) 30:259–69. doi: 10.1016/j.eprac.2023.11.013, 38042448

[3] Borga M West J Bell JD Harvey NC Romu T Heymsfield SB . Advanced body composition assessment: from body mass index to body composition profiling. J Investig Med. (2018) 66:1–9. doi: 10.1136/jim-2018-000722, 29581385 PMC5992366

[4] Neeland IJ Ayers CR Rohatgi AK Turer AT Berry JD Das SR . Associations of visceral and abdominal subcutaneous adipose tissue with markers of cardiac and metabolic risk in obese adults. Obesity. (2013) 21:E439–47. doi: 10.1002/oby.20135, 23687099 PMC3751977

[5] Waters DL. Intermuscular adipose tissue: a brief review of etiology, association with physical function and weight loss in older adults. Ann Geriatr Med Res. (2019) 23:3–8. doi: 10.4235/agmr.19.0001, 32743278 PMC7387605

[6] Chen K Shen Z Gu W Lyu Z Qi X Mu Y . Prevalence of obesity and associated complications in China: a cross-sectional, real-world study in 15.8 million adults. Diabetes Obes Metab. (2023) 25:3390–9. doi: 10.1111/dom.15238, 37589256

[7] Hemmingsson E. The unparalleled rise of obesity in China: a call to action. Int J Obes. (2021) 45:921–2. doi: 10.1038/s41366-021-00774-w, 33608648

[8] Zhao X Sun HY Zhang XD Deng XQ. Analysis of the progress in youth health policies and work since the implementation of the medium- and long-term youth development plan (2016-2025) in China. Chin Youth Stud. (2020) 12:38–47. doi: 10.19633/j.cnki.11-2579/d.2020.0177

[9] Coates AM Joyner MJ Little JP Jones AM Gibala MJ. A perspective on high-intensity interval training for performance and health. Sports Med. (2023) 53:85–96. doi: 10.1007/s40279-023-01938-6, 37804419 PMC10721680

[10] Guo Z Li M Cai J Gong W Liu Y Liu Z. Effect of high-intensity interval training vs. moderate-intensity continuous training on fat loss and cardiorespiratory fitness in the young and middle-aged a systematic review and meta-analysis. Int J Environ Res Public Health. (2023) 20:4741. doi: 10.3390/ijerph20064741, 36981649 PMC10048683

[11] Semenova EA Miyamoto-Mikami E Akimov EB Al-Khelaifi F Murakami H Zempo H . The association of hfe gene h63d polymorphism with endurance athlete status and aerobic capacity: novel findings and a meta-analysis. Eur J Appl Physiol. (2020) 120:665–73. doi: 10.1007/s00421-020-04306-8, 31970519 PMC7042188

[12] Lai JR Gong L Liu Y Li YC Nie J Zhou DQ. Association between adcy3 gene polymorphism and the effects of high-intensity interval training on body composition. Sheng Li Xue Bao. (2024) 76:970–8. doi: 10.13294/j.aps.2024.0062 39780573

[13] Gao S. (2021) Association Study of NNMT and FGF21 gene Polymorphisms with Individual Differences in Health Promotion Effects of HIIT. [Dissertation/Master's thesis]. [Hohhot]: Inner Mongolia Normal University

[14] Marais AD. Apolipoprotein E in lipoprotein metabolism, health and cardiovascular disease. Pathology. (2019) 51:165–76. doi: 10.1016/j.pathol.2018.11.002, 30598326

[15] Zhang Y Cheng Z Hong L Liu J Ma X Wang W . Apolipoprotein E (ApoE) orchestrates adipose tissue inflammation and metabolic disorders through nlrp3 inflammasome. Mol Biomed. (2023) 4:47. doi: 10.1186/s43556-023-00158-8, 38062308 PMC10703753

[16] Shatwan IM Winther KH Ellahi B Elwood P Ben-Shlomo Y Givens I . Association of apolipoprotein E gene polymorphisms with blood lipids and their interaction with dietary factors. Lipids Health Dis. (2018) 17:98. doi: 10.1186/s12944-018-0744-2, 29712557 PMC5928585

[17] Smith EN Chen W Kähönen M Kettunen J Lehtimäki T Peltonen L . Longitudinal genome-wide association of cardiovascular disease risk factors in the Bogalusa heart study. PLoS Genet. (2010) 6:e1001094. doi: 10.1371/journal.pgen.1001094, 20838585 PMC2936521

[18] Yang XL Chu WW Zhou DQ Wu M Liu Y Han M . Association between apolipoprotein E (APOE) gene single nucleotide polymorphisms and sensitivity of blood lipids to high-intensity interval training. Chin J Phys Med Rehabil. (2024) 46:961–6. doi: 10.3760/cma.j.cn421666-20240531-00419

[19] Choi KY Lee JJ Gunasekaran TI Kang S Lee W Jeong J . Apoe promoter polymorphism-219t/g is an effect modifier of the influence of apoe ε4 on alzheimer's disease risk in a multiracial sample. J Clin Med. (2019) 8:1236. doi: 10.3390/jcm8081236, 31426376 PMC6723529

[20] Wu DP. Effect of Rs405509 on Brain Function during Non-demented Aging: A Resting-State Functional Magnetic Resonance Imaging Study. Dissertation/master's thesis. Qingdao: Qingdao University (2022).

[21] Tejedor MT Garcia-Sobreviela MP Ledesma M Arbones-Mainar JM. The apolipoprotein e polymorphism rs7412 associates with body fatness independently of plasma lipids in middle aged men. PLoS One. (2014) 9:e108605. doi: 10.1371/journal.pone.0108605, 25268647 PMC4182517

[22] Huang M Zheng J Chen L You S Huang H. Role of apolipoproteins in the pathogenesis of obesity. Clin Chim Acta. (2023) 545:117359. doi: 10.1016/j.cca.2023.117359, 37086940

[23] Liu K Chen B Zeng F Wang G Wu X Liu Y . Apoe/nos3 knockout mice as a novel cardiovascular disease model of hypertension and atherosclerosis. Genes. (2022) 13:1998. doi: 10.3390/genes13111998, 36360235 PMC9690224

[24] Niimi M Yang D Kitajima S Ning B Wang C Li S . Apoe knockout rabbits: a novel model for the study of human hyperlipidemia. Atherosclerosis. (2016) 245:187–93. doi: 10.1016/j.atherosclerosis.2015.12.002, 26724529

[25] Altenburg MK Maeda N. Apoe4 trapping by the low density lipoprotein receptor, international congress series. Elsevier. (2004) 1262:388–91. doi: 10.1016/j.ics.2003.11.035,

[26] Ozen E Lovegrove JA Jackson KG. Association between body composition and cardiometabolic disease risk: role of dietary fat intake and apolipoprotein e genotype on this relationship. Proc Nutr Soc. (2024) 23:1–9. doi: 10.1017/s0029665124000053 38253522

[27] Chen L Sun L. Bisphenol a exposure and its impact on childhood obesity: a molecular and genetic perspective. Drug Chem Toxicol. (2025) 30:1–11. doi: 10.1080/01480545.2025.2553869, 40884426

[28] Jiang CL Lin FJ. Insights into the roles of apolipoprotein e in adipocyte biology and obesity. Int J Obes. (2024) 48:1205–15. doi: 10.1038/s41366-024-01549-9, 38839985

[29] Xu S Gong L Chu W Zhou D. Effects of 12 weeks of high-intensity interval training on the human gut microbiota. Microbiol China. (2021) 48:1215–26. doi: 10.13344/j.microbiol.china.200676

[30] Berg J Nauman J Wisløff U. Normative values for body composition in 22,191 healthy norwegian adults 20-99 years: the hunt4 study. Prog Cardiovasc Dis. (2024) 85:82–92. doi: 10.1016/j.pcad.2024.06.002, 38925258

[31] Mao B Zhang J Li S Fan Z Deng Y Quan H . Association of body composition with ambulatory blood pressure among chinese youths. BMC Pediatr. (2024) 24:566. doi: 10.1186/s12887-024-05029-x, 39237958 PMC11378592

[32] Zhang Z Tao L Chen Z Zhou D Kan M Zhang D . Association of genetic loci with blood lipids in the Chinese population. PLoS One. (2011) 6:e27305. doi: 10.1371/journal.pone.0027305, 22073310 PMC3207848

[33] Kathiresan S Willer CJ Peloso GM Demissie S Musunuru K Schadt EE . Common variants at 30 loci contribute to polygenic dyslipidemia. Nat Genet. (2009) 41:56–65. doi: 10.1038/ng.291, 19060906 PMC2881676

[34] Komurcu-Bayrak E Onat A Yuzbasiogullari B Mononen N Laaksonen R Kähönen M . The apoe -219g/t and +113g/c polymorphisms affect insulin resistance among turks. Metabolism. (2011) 60:655–63. doi: 10.1016/j.metabol.2010.06.016, 20723945

[35] Van Eck M Herijgers N Yates J Pearce NJ Hoogerbrugge PM Groot PH . Bone marrow transplantation in apolipoprotein e-deficient mice. Effect of apoe gene dosage on serum lipid concentrations, (beta)vldl catabolism, and atherosclerosis. Arterioscler Thromb Vasc Biol. (1997) 17:3117–26. doi: 10.1161/01.atv.17.11.3117, 9409301

[36] Karasik D Kiel DP. Evidence for pleiotropic factors in genetics of the musculoskeletal system. Bone. (2010) 46:1226–37. doi: 10.1016/j.bone.2010.01.382, 20149904 PMC4852133

[37] Albert FW Kruglyak L. The role of regulatory variation in complex traits and disease. Nat Rev Genet. (2015) 16:197–212. doi: 10.1038/nrg3891, 25707927

[38] Saini S Walia GK Sachdeva MP Gupta V. Genetics of obesity and its measures in India. J Genet. (2018) 97:1047–71. doi: 10.1007/s12041-018-0987-8 30262717

[39] Loos RJ. Genetic determinants of common obesity and their value in prediction. Best Pract Res Clin Endocrinol Metab. (2012) 26:211–26. doi: 10.1016/j.beem.2011.11.003, 22498250

[40] Sjúrðarson T Kristiansen J Nordsborg NB Gregersen NO Lydersen LN Grove EL . The angiotensin-converting enzyme I/D polymorphism does not impact training-induced adaptations in exercise capacity in patients with stable coronary artery disease. Sci Rep. (2023) 13:18300. doi: 10.1038/s41598-023-45542-0, 37880303 PMC10600103

[41] Smalinskiene A Petkeviciene J Luksiene D Jureniene K Klumbiene J Lesauskaite V. Association between apoe, scarb1, pparα polymorphisms and serum lipids in a population of lithuanian adults. Lipids Health Dis. (2013) 12:120. doi: 10.1186/1476-511x-12-120, 23919842 PMC3751123

[42] Wang L Liu CY Zhao YY Li LH Sun ZQ Li XN . Correlation study on lipid metabolism and LEPR and APOE gene polymorphisms in children with simple obesity. Chin J Clin Med Women Children. (2007) 4:189–93.

[43] Pandit H Jones NS Rebeck GW. Obesity affects brain cortex gene expression in an apoe genotype and sex dependent manner. Int J Obes. (2024) 48:841–8. doi: 10.1038/s41366-024-01481-y, 38454009 PMC11379128

[44] Li H Li Zhang BH Shi JD. Changes in immune function, blood homeostasis, and antioxidant function in ApoE gene-deficient mice. J Peking Univ. (2001) 4:347–50.

[45] Shen XH Cai W Tang QY Feng Y Wu W. Alterations in oxidative stress levels in nutritionally obese rats. J Hyg Res. (2007) 4:440–2.

[46] Oliveira AN Hood DA. Exercise is mitochondrial medicine for muscle. Sports Med. Health Sci. (2019) 1:11–8. doi: 10.1016/j.smhs.2019.08.008, 35782464 PMC9219266

[47] Azhuvalappil S Prasad R Sahadevan P Pradhan H Rai P Sundarakumar JS. Sex-specific differences in the association between apoe genotype and metabolic syndrome among middle-aged and older rural indians. Metabol Open. (2024) 22:100281. doi: 10.1016/j.metop.2024.100281, 38659620 PMC11035107

[48] Shang Y Mishra A Wang T Wang Y Desai M Chen S . Evidence in support of chromosomal sex influencing plasma based metabolome vs. apoe genotype influencing brain metabolome profile in humanized apoe male and female mice. PLoS One. (2020) 15:e0225392. doi: 10.1371/journal.pone.0225392, 31917799 PMC6952084

[49] Jones NS Watson KQ Rebeck GW. High-fat diet increases gliosis and immediate early gene expression in apoe3 mice, but not apoe4 mice. J Neuroinflammation. (2021) 18:214. doi: 10.1186/s12974-021-02256-2, 34537055 PMC8449905

[50] Sun L Li FH. Effects of long-term high-intensity interval training on the adiponectin/AMPK signaling pathway and cellular autophagy in aging rat skeletal muscle. China Sport Sci. (2018) 38:50–9. doi: 10.16469/j.css.201811005

[51] Lu WP Wen ZF Liu JY Miao PY Chen YL Yang Y . Improvement of salvianolic acid B on nonalcoholic fatty liver disease in ApoE knockout mice and its mechanism. Chin Pharmacol Bull. (2020) 36:31–7.

[52] Ni P Su Y Wang Z Cui J Lu P Li F. Difference analysis of mirna expression profiles in aged female rat adipose tissue regulated by hiit and mict. Cell Biochem Biophys. (2025) 83:3833–44. doi: 10.1007/s12013-025-01757-8, 40246773

[53] Cui XW Li LF Yang XY Xu JF. Effects of acute high-intensity interval exercise and moderate-intensity continuous exercise on postprandial blood glucose, insulin, and inflammatory factors in patients with type 2 diabetes. China Sport Sci. (2022) 42:72–6. doi: 10.16469/j.css.202203007

[54] Ereqat S Cauchi S Eweidat K Elqadi M Ghatass M Sabarneh A . Association of DNA methylation and genetic variations of the apoe gene with the risk of diabetic dyslipidemia. Biomed Rep. (2022) 17:61. doi: 10.3892/br.2022.1544, 35719839 PMC9198989

[55] Radwan ZH Wang X Waqar F Pirim D Niemsiri V Hokanson JE . Comprehensive evaluation of the association of apoe genetic variation with plasma lipoprotein traits in U.S. whites and african blacks. PLoS One. (2014) 9:e114618. doi: 10.1371/journal.pone.0114618, 25502880 PMC4264772

[56] Tao SX. Correlation between Apolipoprotein E Genotype and Promoter Polymorphism Distribution and Coronary Heart Disease in Xinjiang Kazakh Population. [Dissertation/Master's thesis. Ürümqi: Xinjiang Medical University (2015).

[57] Kulminski AM Loika Y Culminskaya I Huang J Arbeev KG Bagley O . Independent associations of tomm40 and apoe variants with body mass index. Aging Cell. (2019) 18:e12869. doi: 10.1111/acel.12869, 30462377 PMC6351823

[58] Zhai W Zhang G Wei C Zhao M Sun L. The obesity paradox in cognitive decline: impact of bmi dynamics and apoe genotypes across various cognitive status. Diabetes Obes Metab. (2025) 27:3967–83. doi: 10.1111/dom.16433, 40317984 PMC12146477

[59] Colton CA Brown CM Vitek MP. Sex steroids, apoe genotype and the innate immune system. Neurobiol Aging. (2005) 26:363–72. doi: 10.1016/j.neurobiolaging.2004.08.001, 15639315

[60] Hellén M Weert I Müller SA Räsänen N Kettunen P Lehtonen Š . Inflammation-induced lysosomal dysfunction in human ipsc-derived microglia is exacerbated by apoe 4/4 genotype. J Neuroinflammation. (2025) 22:147. doi: 10.1186/s12974-025-03470-y, 40457456 PMC12131611

[61] Li Q Shang Y Huang HC Dai X Lao F. The synergistic role of apoe4 and gsk3β in alzheimer's disease: pathological mechanisms and therapeutic implications. Mol Neurobiol. (2025) 63:32. doi: 10.1007/s12035-025-05329-y, 41222729

[62] Laffont I Takahashi M Shibukawa Y Honke K Shuvaev VV Siest G . Apolipoprotein e activates akt pathway in neuro-2a in an isoform-specific manner. Biochem Biophys Res Commun. (2002) 292:83–7. doi: 10.1006/bbrc.2002.6586, 11890675

[63] Huang YA Zhou B Nabet AM Wernig M Südhof TC. Differential signaling mediated by apoe2, apoe3, and apoe4 in human neurons parallels alzheimer's disease risk. J Neurosci. (2019) 39:7408–27. doi: 10.1523/jneurosci.2994-18.2019, 31331998 PMC6759032

[64] Zhao T Fan J Abu-Zaid A Burley SK Zheng XFS. Nuclear mTOR signaling orchestrates transcriptional programs underlying cellular growth and metabolism. Cells. (2024) 13:781. doi: 10.3390/cells13090781, 38727317 PMC11083943

[65] Fan J Khanzada Z Xu Y. Mechanisms underlying muscle-related diseases and aging: insights into pathophysiology and therapeutic strategies. Muscles. (2025) 4:26. doi: 10.3390/muscles4030026, 40843913 PMC12371960

